# Intussusception of Gastrojejunostomy After Pancreatoduodenectomy With Billroth II Reconstruction

**DOI:** 10.7759/cureus.51880

**Published:** 2024-01-08

**Authors:** Alejandro Martinez-Esteban, Natalia M Barron-Cervantes, Pablo Avila-Sanchez, Carlos Chan-Nuñez

**Affiliations:** 1 General and Gastrointestinal Surgery Service, Fundación Clínica Médica Sur, Mexico City, MEX; 2 Department of Hepato-Pancreato-Biliary Surgery, Instituto Nacional de Ciencias Medicas y Nutrición Salvador Zubirán, Mexico City, MEX; 3 Department of Hepato-Pancreato-Biliary Surgery, Instituto Nacional de Ciencias Médicas y Nutrición Salvador Zubirán, Mexico City, MEX

**Keywords:** complication, pancreaticoduodenectomy, cholangiocarcinoma, whipple’s procedure, small bowel obstruction, hepatobiliary, gastrojejunostomy, intussusception

## Abstract

Gastrojejunal anastomosis or gastrojejunostomy (GJ) is a surgical procedure used for allowing gastric emptying, especially in cases where complex reconstructions are needed. One of the less common complications but one of the most relevant in morbidity is the intussusception of the GJ. It requires a high index of suspicion, preoperative optimization of the patient, diagnostic corroboration, and identification of associated complications with the use of contrasted imaging. It was described for the first time by Bozzi in 1914; currently, multiple cases have been described in the literature, being more frequent in bariatric surgeries and reconstructions after distal gastrectomy. In hepatopancreaticobiliary surgery, it is an even uncommon complication. We present the case of a 60-year-old man with intussusception of the efferent loop of the GJ after a pylorus-preserving pancreatoduodenectomy with a Billroth II reconstruction in the setting of malignancy of the extrahepatic bile duct along with our emergency surgical treatment.

## Introduction

Gastrojejunostomy (GJ) anastomosis is a surgical procedure where a new junction is made between the stomach and the jejunum. It is typically performed in either an open or laparoscopic manner, is used to allow gastric emptying where it is not possible to use the anatomical pathway, and can be used as a bypass in cases of obstructions in the distal portion of the stomach or when the extrahepatic biliary tract are affected, such as bariatric and metabolic, upper gastrointestinal, or complex oncological surgeries along with hepatopancreaticobiliary (HPB) surgeries. The first successful GJ to treat gastric outlet obstruction to bypass a cancer of the pylorus was reported from Theodor Billroth’s Clinic in Vienna in 1881 by Anton Wolfler. The creation of a GJ where the stomach is anastomosed to the jejunum without creating a separated pancreaticobiliary limb is known as a Billroth II in honor of his surgical teacher. After a distal gastrectomy or antrectomy, bilioenteric derivation, or other pancreaticoduodenal complex surgery a Billroth I (B-I), Billroth II (B-II), and Roux-en-Y reconstruction methods can be used for upper gastrointestinal anastomosis [1]. One of the less common complications of all mentioned procedures is the intussusception of the GJ, with only a few cases reported. The first case was reported by Bozzi in 1914; currently, over 300 cases have been recorded to date. This case is presented to further expand the knowledge about early diagnosis and present our proposal for surgical management, providing other surgeons options to prevent fatal outcomes. We present the case of a 60-year-old male patient with small bowel obstruction secondary to a GJ intussusception following a pylorus-preserving pancreatoduodenectomy (PPPD) procedure (Traverso-Longmire technique) with Child’s procedure and B-II reconstruction secondary to a distal cholangiocarcinoma in a first-level private HPB surgical center in Mexico City.

## Case presentation

**Figure 1:**
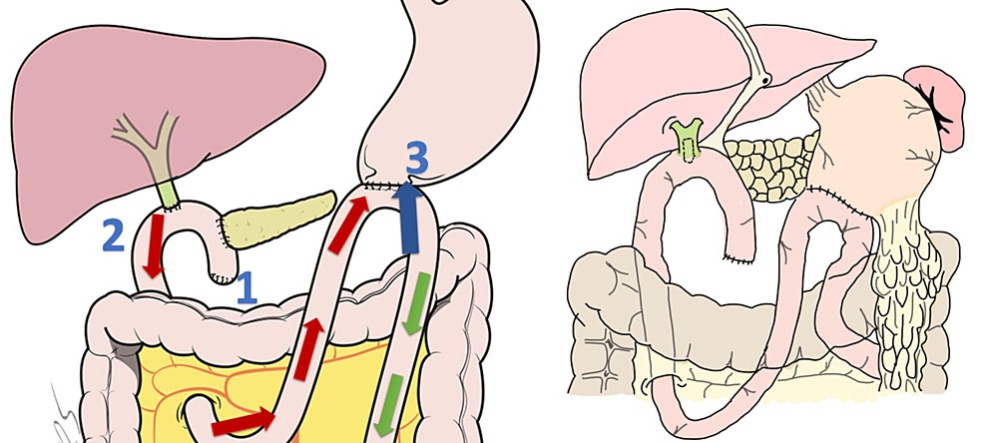
Type II Billroth reconstruction after pancreatoduodenectomy. The classic sequence of Child’s procedure used in this surgery consisted of the following order: (1) retrocolic pancreaticojejunostomy modified Blumgart anastomosis ductal-to-mucosa with external stent; (2) bilioenteric hepaticojejunal end-to-side transmesocolic anastomosis; and (3) gastrojejunal end-to-side antecolic and retrogastric anastomosis with automatic stapler. The afferent loop (red arrow), efferent loop (green arrow), and blue arrow show the direction of intussusception.

A 60-year-old Latin American male patient presented to the emergency room with abdominal pain in the epigastric region, nausea, vomiting, and hematochezia for the last 24 hours. Medical history included the surgical antecedent of having a PPPD, also known as a Traverso-Longmire procedure [2] with a Child’s procedure (Figure 1) [3,4].

During the first months of 2021, the patient presented with jaundice, fever, and pain in the right hypochondrium with a positive Murphy sign. Acute cholangitis was clinically diagnosed, following which an endoscopic retrograde cholangiopancreatography was performed which showed a distal obstruction in the biliary tree reported as a distal cholangiocarcinoma in the biopsy. At the end of 2021, the patient underwent the surgical procedure previously mentioned. For this diagnosis, the surgical procedure was followed by six cycles of adjuvant chemotherapy (gemcitabine + capecitabine + oxaliplatin + irinotecan). The patient was in remission, with the last positron emission tomography-computed tomography scan in October 2022 showing no evidence of abnormal metabolic activity.

In April 2023, he was admitted to the emergency room in a hemodynamically stable condition with a distended, painful abdomen and a palpable mass at the level of the epigastrium and left hypochondrium. During his stay in the emergency department, laboratory findings showed hyperlactatemia (1.4 mmol/L), elevated procalcitonin (2.18 ng/mL), and elevated C-reactive protein (55.5 mg/L) (Table 1).

**Table 1:** Laboratory findings. Laboratory findings from the patient’s stay at the emergency department.

Parameter	Value
Hemoglobin	15.8 g/dL
Hematocrit	46.4%
Platelets	210 × 10^3^/µL
Leukocyte count	6.0 × 10^3^/µL
Absolute neutrophiles	5.5 × 10^3^/µL
Absolute lymphocytes	0.2 × 10^3^/µL
Serum glucose	287 mg/dL
Blood urea nitrogen	24.2 mg/dL
Urea	51.8 mg/dL
Serum creatinine	1.19 mg/dL
Serum Na^+^	138 mmol/L
Serum K^+^	3.6 mmol/L
Serum Cl^-^	107 mmol/L
Lactate	1.4 mmol/L
Procalcitonin	2.18 ng/mL
C-reactive protein	55.5 mg/L
Total bilirubin	1.40 mg/dL
Direct bilirubin	0.24 mg/dL
Indirect bilirubin	1.16 mg/dL
Alanine transaminase	22 U/L
Aspartate transaminase	12 U/L
Lactate dehydrogenase	108 U/L

**Figure 2:**
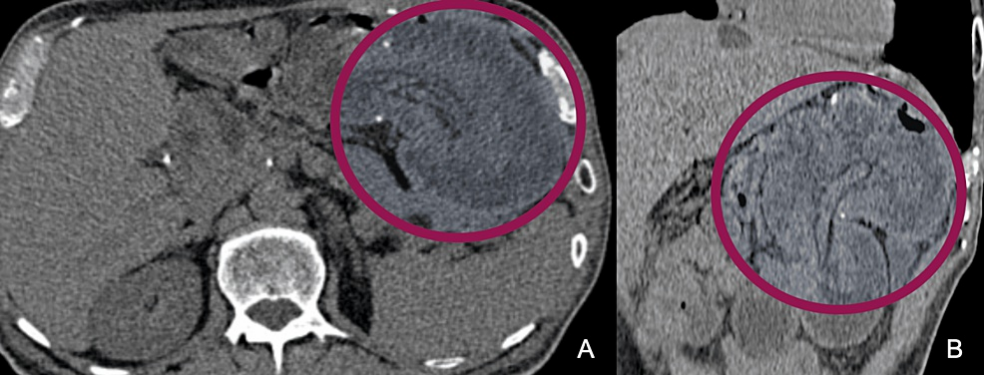
Abdominal CT. Contrasted abdominal axial CT showing intestinal intussusception at the level of the gastrojejunal anastomosis within the stomach (circle), without subdiaphragmatic free air. (A) axial section. (B) Coronal reconstruction.

A CT scan showed a characteristic mass lesion containing fat stripes, suggestive of an intussusception site at the level of the gastrojejunal anastomosis, without evidence of air or free fluid in the abdominal cavity (Figure 2). Throughout his stay, analgesia with acetaminophen 1 g IV and tramadol 50 mg IV were administered and antibiotic therapy with ertapenem 1 g was initiated. The placement of a nasogastric tube was required, which immediately presented expenditure of bile contents. The patient underwent an emergency exploratory laparotomy.

**Figure 3:**
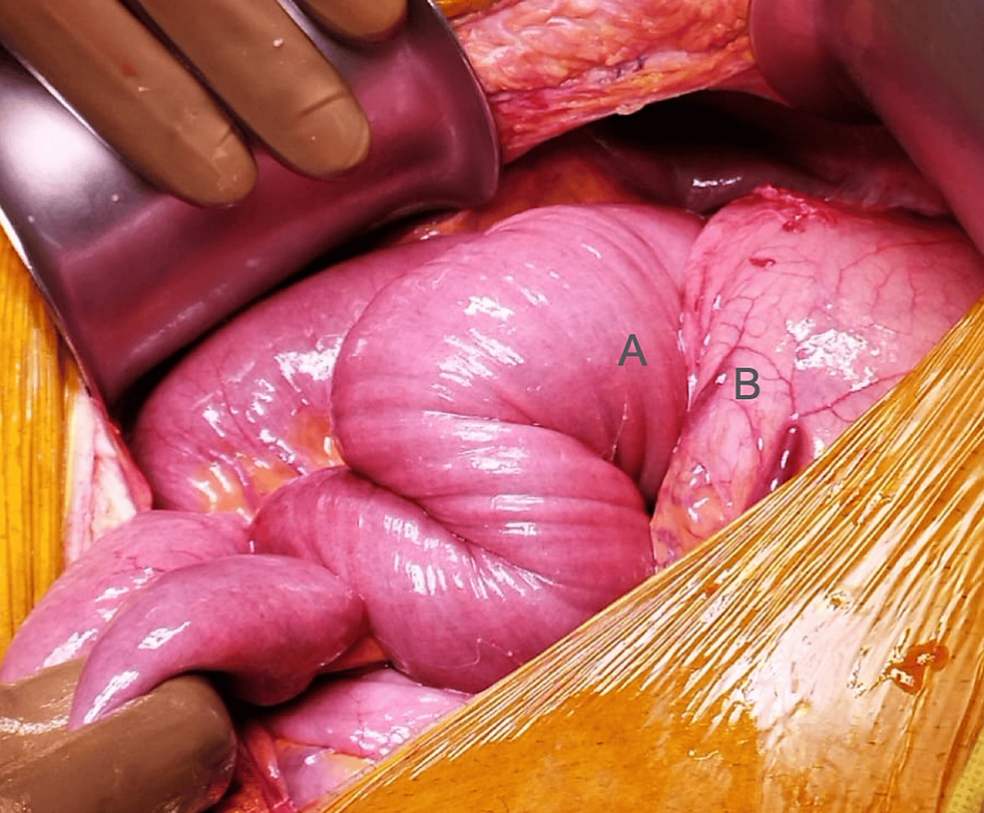
Site of the intussusception. Invagination of the efferent loop (A) into the stomach (B) through the jejunogastric anastomosis Brynits 2A/Shackman 2A.

During the surgery, gastric distension at the expense of invagination of the efferent loop into the stomach through the jejunogastric anastomosis was observed, which was classified as Brynits and Rubinstein 2A/Shackman II (Figure 3) [5].

**Figure 4:**
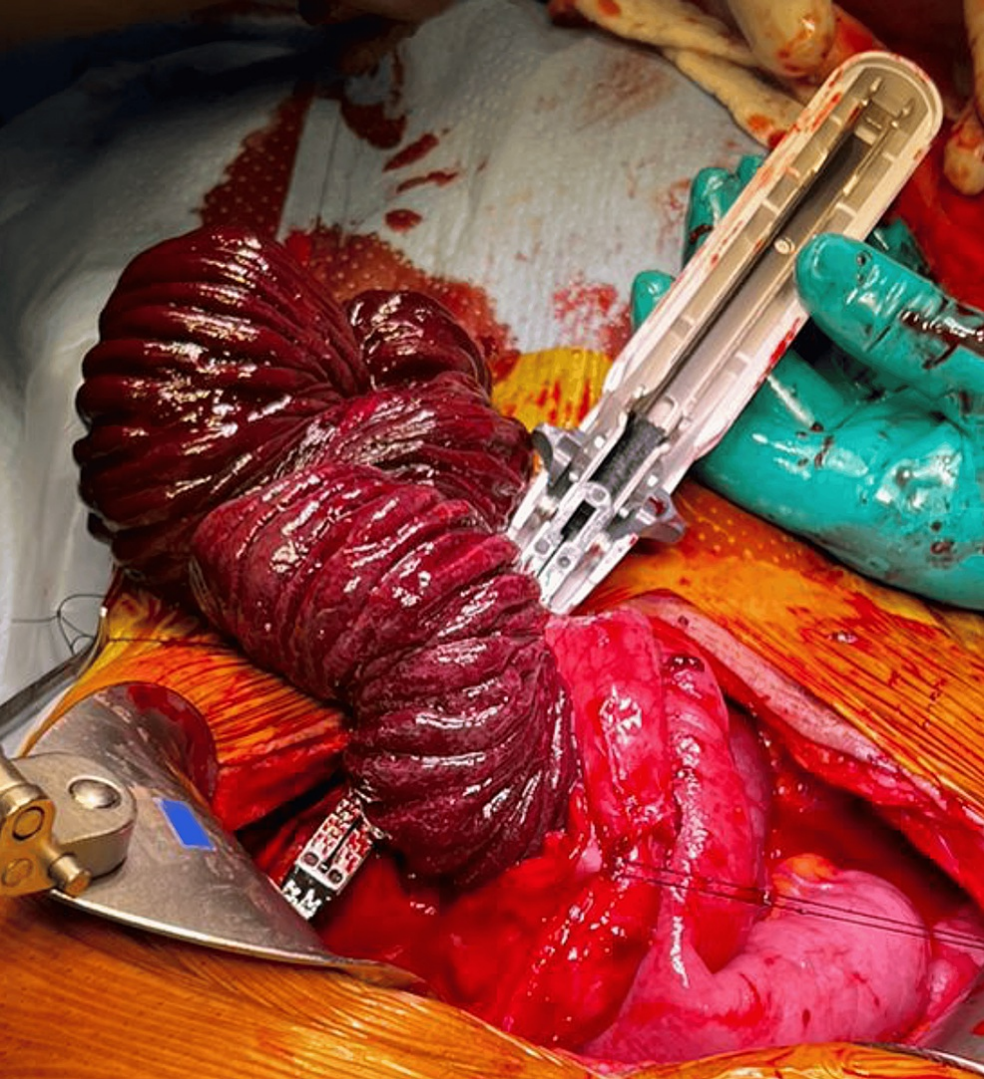
Efferent loop. Middle jejunum with a necrotic appearance.

**Figure 5:**
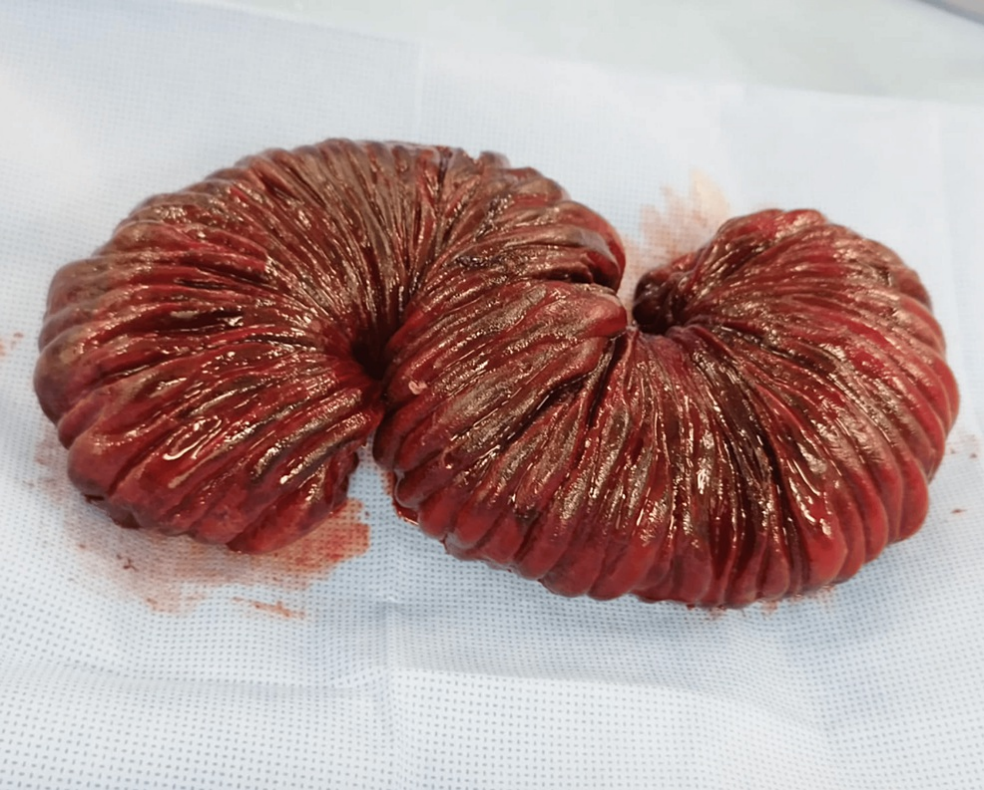
Necrotic middle jejunum. Macroscopic vision of the necrotic middle jejunum.

Hutchinson maneuver was tried but was unsuccessful because of the edema present at the time of the surgery; hence, a gastrostomy of the anterior wall and a transgastric intestinal exteriorization was realized with a mechanical resection of 45 cm of the middle jejunum, which showed a congested and devascularized appearance and vascular compromise of the mesentery (Figures 4, 5). Finally, intestinal transit was restored with a double-layer end-to-end jejunum-jejunum hand-sewn anastomosis and primary gastric repair [6]. During his in-hospital stay, he had an adequate postoperative evolution and was discharged after five days, without any incidents.

## Discussion

For a long time, it was believed that the only curative option for distal cholangiocarcinoma was a liver transplant; however, emerging new surgical techniques have allowed patients to live a healthy life even after the diagnosis. One of the surgical interventions offered to patients with distal cholangiocarcinoma is the modified Whipple’s procedure (Traverso-Longmire technique). PPancreaticoduodenectomy is still a surgical procedure associated with high morbidity and mortality rates; however, the PD procedure has undergone a remarkable gradual evolution in the last 20 years, of which many surgeons are unaware. During this surgical procedure, a duct-to-mucosa pancreaticojejunostomy with a modified Blumgart technique, transmesocolic end-to-lateral hand sewn hepaticojejunostomy as well as a mechanical lateral-to-lateral antecolic, retrogastric and isoperistaltic gastrojejunostomy are made. The forbidding mortality of PD, approximately 20% just a generation ago, has decreased in American high-volume referral centers to near-zero mortality rates [7]. Further, pylorus-sparing procedures may reduce postoperative complications. Its most common complication nowadays is anastomosis leakage; however, it is not the only complication that must be ruled out [8].

**Figure 6:**
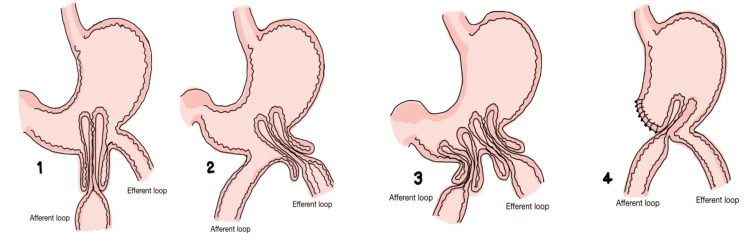
Gastrojejunal anastomosis intussusception Shackman classification. Type I (1): afferent loop. Type II (2): efferent loop. Type III (3): afferent and efferent loop separated by the gastric wall. Type IV (4): afferent and efferent loop inside the same intussusception, no gastric wall between the two loops. Adapted from Abu-Omar et al. [13]. This article is available under the Creative Commons CC-BY-NC license and permits non-commercial use, distribution, and reproduction in any medium, provided the original work is properly cited.

The intussusception of the GJ in PPPD with a B-II reconstruction is a very rare, yet fatal, complication. However, with early identification and proper diagnosis, early surgical treatment may be offered with favorable outcomes, like in this case. It is crucial to emphasize that among the few cases reported, there is another case in which the diagnosis and reason for performing the Longmire-Traverso procedure was distal cholangiocarcinoma; moreover, in all reported cases, the need for surgical treatment has been described [9-11]. Four types have been described in the literature with the most common being the intussusception of the efferent loop of the GJ. Other types are the intussusception of the afferent loop and of both the afferent and efferent loops. The worst-case scenario is the intussusception of both afferent and efferent loops as both loops occupy the same space causing more edema and necrosis can appear earlier than in cases where only one strand is compromised. Surgically, more tissue may need to be resected secondary to this, leading to other complications such as short bowel syndrome (Figure 6) [12].

We opted for a primary reduction of the involved segment; however, due to the significant edema and vascular congestion at the anastomosis site, this was not possible; hence, we decided to perform an anterior gastrotomy with a scalpel and transgastric intestinal exteriorization and resection of the affected portion of jejunum to subsequently perform an end-to-end jejunum-jejunum hand-sewn anastomosis of the afferent loop. We believe that performing a jejunogastric anastomosis using the same afferent and efferent loop placed in an antiperistaltic manner was the mechanism by which intussusception developed. Additionally, as there are very few cases reported in the literature regarding jejunogastric anastomosis intussusception after a PD procedure, the presence of other factors involved may explain the development of this extremely rare complication in this surgical technique.

It is important to mention the importance of imaging studies, such as CT scans, within the approach of acute abdomen in postoperative patients, especially with previous abdominal surgeries that can alter the physiological anatomy and whose pain may present abnormally. Abdominal CT with intravenous contrast is the reference imaging study to establish the diagnosis, the cause, the anatomy, the transition zone, the detection of ischemia, and complications such as perforation. Likewise, it is important to note that in this case, only a small segment of the intestine had to be resected secondary to intestinal necrosis owing to the correct diagnostic approach, which not only avoided mortality but also minimized significant morbidity of our patient.

## Conclusions

In this case, a timely diagnosis and correct clinical approach allowed the identification of jejunogastric intussusception, avoiding a fatal outcome for the patient. Physical examination is crucial to establish a diagnostic suspicion of such a complication. This case proves that this complication should always be suspected in patients who have undergone any type of HPB surgery or upper gastrointestinal reconstruction surgery. Patients who present with symptoms compatible with small bowel obstruction should always undergo a contrasted abdominal CT scan to evaluate the obstruction the intestinal viability to avoid intestinal ischemia and its associated complications. This case report not only describes the possibility of encountering this rare complication in even the best surgical centers in the country in the hands of expert surgeons in the field but also allows the identification of certain risk factors, such as age, viability of the tissue, and anastomosis placement, that may be preventable on future occasions. Emphasizing the importance of early identification and diagnosis of GJ intussusception is crucial in this case. It is also of great significance to draw attention to the fact that a prompt surgical intervention was the key to achieving a good outcome for the patient.
